# Associations Between miR-145, TGF-β Signaling, and Aortic Wall Remodeling in Abdominal Aortic Aneurysm

**DOI:** 10.3390/genes17101183

**Published:** 2026-09-24

**Authors:** Erhan Kaya, Hidayet Demir, Mehrdad Sheikhvatan

**Affiliations:** 1 Özel Pendik Bölge Hastanesi, Istanbul 34890, Turkey; 2 Memorial Göztepe Hospital, Istanbul 34634, Turkey; 3 Department of Medical Biology and Genetics, Istanbul Okan University, Istanbul 34959, Turkey; 4 Department of Medical Genetics, Tehran University of Medical Sciences, Tehran 14176, Iran

**Keywords:** abdominal aortic aneurysm, microRNA-145, transforming growth factor-β

## Abstract

**Background/purpose:** Abdominal aortic aneurysm (AAA) is characterized by pathological remodeling of the aortic wall, involving vascular smooth muscle cell (VSMC) dysfunction, extracellular matrix degradation, and alterations in signaling pathways. MicroRNA-145 (miR-145) is involved in the regulation of VSMC phenotype, whereas transforming growth factor-beta (TGF-β) signaling has context-dependent effects on vascular remodeling. This study used a dataset to explore potential associations between miR-145 expression, TGF-β/SMAD signaling, VSMC phenotypic characteristics, and structural features of the aortic wall. **Methods**: Aortic tissue samples were obtained from 324 patients with AAA during aneurysm-related surgical treatment. Control tissues were collected from macroscopically healthy aortic segments of 324 subjects undergoing elective cardiothoracic or vascular surgery. Tissue samples were analyzed for miR-145 expression, TGF-β signaling, vascular smooth muscle cell markers, and histopathological characteristics of aortic wall remodeling. **Results**: miR-145 was significantly downregulated in AAA tissue compared to control. RT-qPCR, Western blotting, and immunohistochemistry revealed increased TGF-β1/TGFBR signaling, SMAD2/3 activation, and KLF4 expression, accompanied by reduced expression of the VSMC contractile genes ACTA2, TAGLN, and MYH11, in AAA tissue. The lower expression level of miR-145 correlated significantly with TGF-β/SMAD pathway activation, VSMC phenotypic switching, more pronounced medial degeneration and elastic-fiber fragmentation, and increased aortic diameter (*p* < 0.001). **Conclusions**: Lower miR-145 expression was associated with TGF-β/SMAD pathway-related activity, altered VSMC phenotype, and structural remodeling of the aortic wall in AAA tissue. These findings identify a potential association between miR-145 dysregulation and TGF-β-associated vascular remodeling; however, they do not establish a direct functional signaling axis or causal relationship. Further mechanistic studies are required to clarify the underlying molecular interactions.

## 1. Introduction

Abdominal aortic aneurysm (AAA) is a pathological dilatation of the abdominal aorta, generally defined as an aortic diameter of ≥3.0 cm or at least 1.5 times the expected normal diameter [1]. AAA is a clinically important vascular condition because progressive weakening of the aortic wall can result in aneurysm expansion, rupture, life-threatening internal hemorrhage, and death. The global prevalence of AAA among individuals aged 30–79 years was estimated at approximately 0.92% (95% CI: 0.65–1.30%) in 2019, although prevalence varies according to age, sex, smoking history, geographical region, and diagnostic criteria [2]. Older age, male sex, smoking, and family history are important risk factors for AAA development. The biological mechanisms underlying AAA involve extracellular matrix degradation, vascular smooth muscle cell (VSMC) dysfunction and apoptosis, inflammatory responses, and progressive aortic wall remodeling. Ruptured AAA is associated with particularly high mortality, with many patients dying before reaching the hospital [3,4]. Therefore, improved understanding of the molecular processes involved in aortic wall degeneration and remodeling is essential for identifying potential biomarkers and therapeutic targets for disease progression and rupture prevention.

Signaling via transforming growth factor-β (TGF-β) is a key modulator of vascular homeostasis and the integrity of the aortic wall [5]. Using the SMAD-dependent and -independent pathways of action, TGF-β impacts VSMC differentiation, proliferation, extracellular matrix production, and turnover [6]. Disruption in TGF-β signaling is known to be involved in the development of aortic aneurysms and dissections; in fact, evidence shows that modifications in TGF-β signaling specific for VSMCs could lead to impaired mechanical characteristics of the aortic wall and remodeling of its structure [7,8]. It is particularly important that TGF-β signaling is tightly associated with regulation of the VSMC phenotypic processes; thus, disruption in the former could be the cause of remodeled aortic wall [9].

MicroRNAs (miRNAs) have become important post-transcriptional mediators of vascular biology and aortic disease. One such miRNA that plays a very significant role due to its high expression in vascular smooth muscle and contribution to the maintenance of contractile phenotype of VSMC is miR-145 [10]. MiR-145 modulates different transcriptional pathways involved in VSMC differentiation, including those mediated by myocardin, Kruppel-like factors, and other elements of the smooth muscle cell differentiation [11]. The decrease in the expression of miR-145 has been linked to the switch in the VSMC phenotype, proliferation, migration, and vascular remodeling [12]. Mechanical stress is an important factor in vascular smooth muscle cell (VSMC) phenotypic modulation and may contribute to pathological vascular remodeling. In an experimental study, mechanical stretch reduced miR-145 expression in cultured human aortic smooth muscle cells and was accompanied by decreased expression of VSMC contractile markers. The authors reported that pharmacological inhibition of ERK1/2 partially attenuated stretch-induced miR-145 suppression, suggesting that ERK1/2 signaling may be involved in this regulatory process. In addition, mechanical stretch increased angiotensin-converting enzyme (ACE) expression and angiotensin II levels, whereas ACE silencing partially restored miR-145 expression, indicating a possible contribution of ACE-related signaling. These findings suggest that mechanical forces may influence miR-145 expression through ERK1/2- and ACE-associated mechanisms, thereby potentially affecting VSMC phenotype. However, these mechanisms were primarily investigated in an in vitro stretch model and should not be considered directly established in human abdominal aortic aneurysm (AAA) tissue. Further studies are required to determine whether similar regulatory interactions occur during AAA development and progression [13].

The connection between miR-145 and TGF-β signaling seems to be especially significant in relation to aortic remodeling. In this respect, experimental data show that miR-145 is able to affect the TGF-β signaling pathway through the regulation of SMAD4 and subsequent SMAD2/3 signaling and affecting VSMC proliferation and migration [14]. The disruption of the miR-143/145 cluster is connected with activation of TGF-β1 signaling, VSMC phenotype transformation, and aortic media degeneration in AAA tissue. The human aortic aneurysm samples have shown the reduction in miR-143/145 levels, as well as the increase in the phosphorylation of SMAD2/3 and ERK proteins, which confirms the functional role of the mentioned interaction [15]. The miR-143/145 cluster plays an important role in maintaining the contractile phenotype of vascular smooth muscle cells (VSMCs). Evidence from aortic dissection research suggests that reduced expression of this cluster is associated with VSMC phenotypic switching and degeneration of the aortic media. In a study by Zhang and Wang, miR-143 and miR-145 expression was decreased in aortic dissection tissues, whereas phosphorylated SMAD2/3 and ERK expression was increased. Furthermore, experimental knockdown of the miR-143/145 cluster in VSMCs was associated with phenotypic changes and alterations in TGF-β1 signaling. These findings suggest a potential relationship between miR-143/145 dysregulation, TGF-β1-related signaling, and vascular wall degeneration. In an in vitro model using cultured vascular smooth muscle cells (VSMCs), miR-145 overexpression was associated with increased TGF-β1 expression and upregulation of osteopontin (OPN) and collagen type III. Inhibition of the TGF-β1 signaling pathway reduced the effects of miR-145 on OPN and collagen III expression, suggesting a potential role for TGF-β1-related signaling in this response. These findings should be interpreted as evidence of an association within a cellular experimental model rather than definitive proof of a causal pathway in human AAA tissue [16].

Further evidence for miR-145 involvement in AAA provides additional support for this hypothesis. Specifically, expression of miR-145 has been shown to be lower in samples of AAA, and its experimental increase alleviated VSMC phenotypic switching upon mechanical stretching and regulated KLF4 expression [17]. In an in vitro experimental model, mechanical stretching of cultured human aortic VSMCs was reported to reduce miR-145 expression, potentially through ERK1/2- and angiotensin-converting enzyme (ACE)-related signaling mechanisms. Mechanical forces may influence miR-145 expression in VSMCs. In an in vitro experimental model, cultured human aortic smooth muscle cells were subjected to cyclic mechanical stretch, which was associated with reduced miR-145 expression and alterations in the expression of VSMC contractile markers. The reported findings further suggested that ERK1/2 signaling and angiotensin-converting enzyme (ACE)-related mechanisms may contribute to this response. Although these observations provide evidence that mechanical stimulation can affect miR-145 regulation in VSMCs, their relevance to miR-145 dysregulation in human abdominal aortic aneurysm (AAA) tissue remains to be established.

Recently, it has also been demonstrated experimentally that the decrease in the miR-143/145 pathway is accompanied by extracellular matrix softening, phenotypic switching, and AAA, thus pointing to a vicious circle formed by changes in the mechanical properties of the aorta and miRNA-mediated cell response [18]. Recently, Ye et al. demonstrated in vitro, using vascular smooth muscle cells cultured in extracellular matrices with different stiffness levels, that extracellular matrix softening was associated with downregulation of miR-143/145 through AKT signaling and VSMC phenotypic switching. In vivo experiments using a BAPN-induced thoracic aortic dissection mouse model further showed that miR-143/145 overexpression attenuated aortic softening, VSMC phenotypic switching, and TAD development. These findings suggest a potential relationship between extracellular matrix mechanical properties, miR-143/145 regulation, and aortic wall pathology; however, their direct relevance to AAA remains to be established [19].

However, the interaction between the expression of miR-145, the TGF-β pathway, and the aortic wall remodeling in AAA has not been sufficiently studied. The problem is that it is not completely clear whether changes in miR-145 expression have any connection to certain characteristics of the aortic wall remodeling and whether the connection is compatible with the suggested function of the TGF-β pathway in VSMC phenotype maintenance and ECM integrity. Understanding this connection may help to reveal the molecular mechanisms behind aortic wall vulnerability and the nature of pathological remodeling rather than diagnosing the disease.

Thus, the objective of this study was to investigate the association between miR-145 expression and biomarkers related to aortic wall remodeling, including components of the TGF-β/SMAD signaling pathway and vascular smooth muscle cell phenotype, in patients with aortic dissection. The study further aimed to characterize the potential relationship between miR-145 expression and TGF-β/SMAD-related molecular alterations, generating hypotheses for future mechanistic investigations.

## 2. Materials and Methods

### 2.1. Study Design and Patient Population

The current observational case–control study was aimed at exploring the relationship between miR-145 expression, TGF-β signaling pathway, and aortic wall structure remodeling in patients with AAA. Patients with AAA who underwent open surgical repair at the cardiovascular surgery department of Private Pendik Regional Hospital between September 2019 and December 2025 were recruited for the study. Thus, retrospective sampling accounted for 100% of the analyzed archived specimens. The Institutional Ethics Committee determined that the use of previously collected, anonymized archived materials for retrospective research did not require additional informed consent, provided that no additional procedures or prospective specimen collection were performed. The archived specimens were linked to the corresponding clinical records through study-specific codes, while direct patient identifiers were removed from the research dataset. The predefined clinical variables required for the study were available through the institutional medical records/database and were analyzed in conjunction with the corresponding biological specimens. The diagnosis of AAA was established based on the clinical presentation and cross-sectional vascular imaging using contrast-enhanced computed tomography angiography (CTA), in accordance with established clinical and radiological criteria. AAA was defined as a focal dilatation of the infrarenal abdominal aorta with a maximum outer-to-outer diameter of ≥3.0 cm (30 mm) or at least 1.5 times the expected normal aortic diameter. CTA assessment included measurement of the maximum aortic diameter perpendicular to the vessel’s longitudinal axis, together with evaluation of aneurysm morphology, mural thrombus, aortic wall characteristics, and potential complications, including dissection, contained rupture, or rupture. Measurements were obtained using standardized imaging planes to minimize errors related to oblique or non-orthogonal measurements. The control group comprised 324 patients undergoing clinically indicated open cardiovascular surgical procedures during which an aortic segment was directly exposed and from which a small portion of macroscopically healthy aortic wall could be obtained without additional operative intervention.

The primary objective was to determine whether miR-145 expression differed between AAA and control aortic tissue. Secondary objectives were to evaluate the relationship between miR-145 expression and components of the TGF-β signaling pathway, vascular smooth muscle cell (VSMC) phenotype, and histopathological indicators of aortic wall remodeling.

### 2.2. Inclusion and Exclusion Criteria

We included patients who were at least 18 years old, had imaging that clearly showed AAA, had enough aortic tissue or blood samples for molecular testing, and had all the necessary clinical and imaging data. The exclusion criteria were a history of connective tissue disorders that affect the aorta, previous AAA or major aortic surgery (involved a major surgical procedure—typically requiring a thoracotomy (chest incision), laparotomy (abdominal incision), or sternotomy (breastbone split)—to repair, replace, or reinforce a diseased segment of the thoracic or abdominal aorta), active cancer, ongoing inflammatory or infectious diseases, or if their tissue samples were not good enough for detailed analysis. Participants were excluded if sufficient clinical information was unavailable or if their RNA samples failed to meet the predefined quality-control criteria. For the control group, individuals with a known aortic aneurysm, AAA, significant aortic atherosclerotic disease, active systemic inflammation, or other conditions potentially affecting the biological characteristics of the aortic wall were excluded.

### 2.3. Clinical and Demographic Data Collection

Demographic and clinical data were extracted from medical records using a standardized data-collection form. The variables collected included age, sex, body mass index (BMI), smoking status, hypertension, diabetes mellitus, dyslipidemia, coronary artery disease, chronic kidney disease, and overall cardiovascular history. Pre-admission and preoperative medication use was also documented, including antihypertensive agents, statins, antiplatelet drugs, anticoagulants, and other cardiovascular medications. For patients with AAA, additional disease-specific variables were recorded, including aneurysm location and extent, maximum aortic diameter, involvement of major branch vessels, the presence of intramural hematoma or penetrating aortic ulcer, and the interval between symptom onset and tissue collection. When available, imaging studies were independently reviewed by one or two experienced cardiovascular radiologists who were blinded to the molecular findings.

### 2.4. Definitions of Clinical Variables

Hypertension was defined as a previously documented diagnosis of hypertension, current use of antihypertensive medication, or blood pressure measurements meeting the prespecified diagnostic threshold according to contemporary clinical guidelines [20]. Diabetes mellitus was defined as a previous physician diagnosis, current use of glucose-lowering medication, or laboratory evidence fulfilling accepted diagnostic criteria [21]. Dyslipidemia was defined as a previous diagnosis, current lipid-lowering therapy, or lipid concentrations meeting the prespecified diagnostic thresholds [22]. Smoking status was categorized as never-smoker, former smoker, or current smoker. A current smoker was defined as an individual who was actively smoking at the time of enrollment, whereas former smokers were defined as individuals who had previously smoked but had stopped smoking before enrollment. The time elapsed since smoking cessation was not systematically recorded and was therefore not included in the analysis.

### 2.5. Blood Sampling and Biochemical Assessment

Peripheral blood samples were drawn into tubes containing EDTA for plasma extraction and, whenever possible, prior to any surgical interventions and transfusions. As part of routine clinical practice, the following laboratory tests were performed in the laboratory of Private Pendik Regional Hospital: blood cell count, serum creatinine, total cholesterol, blood glucose level, HDL cholesterol, LDL cholesterol, triglycerides, and inflammatory markers including C-reactive protein. To perform molecular analyses, plasma was obtained by centrifugation of blood samples, and the plasma obtained was properly transferred to laboratory tubes without disturbance of the blood cells. Plasma produced was frozen immediately after the procedure at the temperature of −80 °C for further extraction of RNA and assessment of miR-145 level. Prior to extraction of RNA, samples were thawed at room temperature and processed using RNase-free techniques as described below. Before RNA extraction, frozen plasma samples were removed from −80 °C storage and thawed at room temperature under controlled laboratory conditions. To minimize RNA degradation and potential contamination, all sample handling procedures were performed using RNase-free consumables, certified RNase-free pipette tips, and clean, designated work surfaces. Laboratory personnel wore disposable gloves and changed them regularly to reduce the risk of RNase contamination. Sample tubes were handled individually, and unnecessary exposure to ambient conditions was minimized.

Following thawing, samples were gently mixed by pipetting or brief, low-speed vortexing, as appropriate, to ensure sample homogeneity while avoiding excessive agitation. Samples were visually inspected for abnormalities, including substantial precipitation or contamination, and were processed promptly after thawing. Where necessary, samples were briefly centrifuged to collect the contents at the bottom of the tube and remove droplets from the tube walls. The processed samples were then used for RNA extraction according to the manufacturer’s instructions for the selected RNA isolation kit. The number of freeze–thaw cycles was recorded where available, and repeated freeze–thawing was avoided whenever possible.

### 2.6. Aortic Tissue Collection and Processing

The tissue of aortic walls was collected during open surgical repair from the section of dissected aorta. Aortic tissue samples were collected from the aneurysmal sac, preferably from the region of maximum aortic dilatation, to ensure that the specimens were representative of the diseased aortic wall. Control tissue samples were obtained from macroscopically healthy aortic segments corresponding to the same anatomical region, whenever feasible. Control specimens were collected only from surgically exposed aortic tissue and were not obtained from patients undergoing isolated peripheral vascular procedures in which the aorta was not exposed. The specimens were selected from areas without macroscopic evidence of aneurysm, dissection, or significant atherosclerotic disease and, whenever anatomically feasible, corresponded to the same aortic region represented in the AAA specimens. Tissue collection was performed by the operating surgeon during the clinically indicated procedure and did not require an additional surgical procedure for research purposes. Control tissues were treated in compliance with all the protocols to minimize the level of variability before the analysis was performed. Immediately after collection, the tissue samples were divided into separate portions for molecular and histological analyses. Samples designated for molecular analysis were rinsed with sterile saline, immediately transferred into RNase-free containers, and stored under appropriate conditions for subsequent RNA extraction and analysis.

### 2.7. Histopathological Assessment of Aortic Wall Remodeling

The structural changes in the aorta wall were documented using histological image analysis. The examination was done through the application of the hematoxylin and eosin (H&E) staining technique in order to produce pictures of the tissue architecture. In addition to H&E staining, other types of staining were utilized to see the changes in the extracellular matrix that could concern collagen; Masson’s trichrome and Verhoeff–Van Gieson/Elastica van Gieson staining produced images of the state of the elastic fibers. The histopathological parameters of the aorta include: degeneration of the middle layer, loss and fragmentation of elastic lamellae, collagen deposition, disordering of smooth muscle cells, infiltration of inflammatory cells, and presence of necrosis and hemorrhages. When appropriate, each of the proposed parameters was evaluated through a certain semiquantitative scale by two experts who had no information about molecular indicators. In the case of disagreements, consensus or independent evaluation was applied.

### 2.8. Immunohistochemical Assessment of VSMC and TGF-β Signaling Markers

Immunohistochemical techniques were utilized to stain aortic tissues treated to keep them intact and later embedded in paraffin. These staining techniques used antibodies that had been validated to confirm the features of TGF-β signaling as well as the VSMC phenotype. The primary antibodies used for immunohistochemical staining included antibodies against α-smooth muscle actin (α-SMA/ACTA2), SM22α/transgelin (SM22α/TAGLN), calponin (CNN1), transforming growth factor-β1 (TGF-β1), transforming growth factor-β receptor II (TGFBR2), total SMAD2, phosphorylated SMAD2 (p-SMAD2), total SMAD3, and phosphorylated SMAD3 (p-SMAD3). Detailed information regarding the antibodies, including the manufacturer, catalog number, host species, clone number (where applicable), working dilution, and RRID (where available), is provided in Table 1.

Aortic tissue sections were deparaffinized in xylene and rehydrated using a graded ethanol series. Heat-induced antigen retrieval was performed by immersing the sections in sodium citrate buffer and heating them at 95–100 °C for 20 min. After cooling to room temperature, the sections were rinsed with phosphate-buffered saline (PBS). Endogenous peroxidase activity was blocked by treating the sections with hydrogen peroxide (H_2_O_2_) for 10 min at room temperature. The sections were subsequently washed three times with PBS and incubated overnight at 4 °C with the primary antibodies.

Immunohistochemical images were acquired using an Olympus BX53 light microscope equipped with a DP74 digital camera (Olympus Corporation, Tokyo, Japan) at ×100 and ×400 magnifications. For each specimen, five non-overlapping representative fields within the aortic tunica media were randomly selected and photographed using identical illumination, exposure, and magnification settings. Quantitative image analysis was performed using ImageJ software (National Institutes of Health, Bethesda, MD, USA; version 1.54) [23]. The percentage of positively stained area and staining intensity were quantified within predefined regions of the tunica media for each field, and the mean values were calculated for each specimen. Image acquisition and quantitative assessments were performed by an investigator blinded to the clinical and molecular characteristics of the study groups.

### 2.9. RNA Extraction from Aortic Tissue

Total RNA was extracted from the aortic tissue samples using the RNeasy Fibrous Tissue Mini Kit (QIAGEN, Hilden, Germany; Cat. No. 74704) according to the manufacturer’s instructions. Aortic tissue samples (approximately 20–50 mg) were mechanically homogenized under RNase-free conditions using a TissueRuptor II handheld rotor–stator homogenizer (QIAGEN, Hilden, Germany; catalog no. 9002755) in QIAzol Lysis Reagent supplied with the miRNeasy Mini Kit (QIAGEN, Hilden, Germany; catalog no. 217004). Total RNA, including small RNA species, was subsequently extracted according to the manufacturer’s instructions. DNase treatment was performed when necessary to minimize genomic DNA contamination. The procedure was conducted in accordance with the manufacturers’ instructions. To minimize potential genomic DNA contamination, extracted RNA was treated with RNase-free DNase I (QIAGEN, Hilden, Germany; catalog number to be verified) according to the manufacturer’s instructions. Briefly, the RNA samples were incubated with DNase I in the recommended reaction buffer under the specified incubation conditions. Following treatment, the enzyme was removed or inactivated according to the manufacturer’s protocol, and the RNA was subsequently used for downstream analysis. The concentration of RNA obtained from this process was checked through the use of microvolume spectrophotometer, whereby the ratios A260/A280 and A260/A230 were then noted down accordingly. The concentration and purity of the extracted RNA were assessed using a NanoDrop One Microvolume UV–Vis Spectrophotometer (Thermo Fisher Scientific, Waltham, MA, USA). The instrument was blanked using RNase-free water before measurement. An aliquot of each RNA sample was applied to the measurement pedestal, and absorbance at 260 nm was used to determine RNA concentration, which was expressed in ng/µL. RNA purity was evaluated using the A260/A280 and A260/A230 absorbance ratios, as calculated by the instrument. RNA integrity was assessed using an Agilent 2100 Bioanalyzer with the RNA 6000 Nano Kit (Agilent Technologies, Santa Clara, CA, USA). Samples with an RNA Integrity Number (RIN) below 7.0 were considered to have substantial RNA degradation and were excluded from subsequent molecular analyses. Samples with a RIN of ≥7.0 were considered acceptable for downstream analysis.

### 2.10. Quantification of miR-145 Expression

Expression of mature miR-145 was assessed using reverse-transcription quantitative polymerase chain reaction (RT-qPCR). Total RNA, including small RNA, was extracted from approximately 50 mg of frozen aortic tissue using the miRNeasy Mini Kit (QIAGEN, Hilden, Germany), according to the manufacturer’s instructions. RNA concentration and purity were assessed using a NanoDrop spectrophotometer (Thermo Fisher Scientific, Waltham, MA, USA). For reverse transcription, 500 ng of total RNA from each sample was used in a final reaction volume of 20 μL with the miScript II RT Kit (QIAGEN), according to the manufacturer’s protocol. The reverse-transcription reaction was performed using the miRNA-specific primer system provided by the kit to facilitate the conversion of mature miRNAs into complementary DNA (cDNA). Quantification of mature miR-145 was performed using a miR-145-specific miScript Primer Assay and the miScript SYBR Green PCR Kit (QIAGEN). Amplification was conducted using a Rotor-Gene Q real-time PCR cycler (QIAGEN). Each qPCR reaction was performed in duplicate in a final volume of 20 μL. The reaction mixture contained 10 μL of 2× miScript SYBR Green PCR Master Mix, 2 μL of diluted cDNA, 2 μL of the specific primer assay, and nuclease-free water to achieve the final reaction volume. The thermal cycling protocol consisted of an initial activation step at 95 °C for 15 min, followed by 40 amplification cycles of denaturation at 94 °C for 15 s, annealing at 55 °C for 30 s, and extension at 70 °C for 30 s. Melting-curve analysis was performed after amplification to assess amplification specificity and identify potential nonspecific products or primer dimers. No-template controls containing nuclease-free water instead of cDNA were included in each analytical run to monitor potential reagent contamination. Reverse-transcription-negative controls were also included when appropriate to assess possible amplification from genomic DNA or other non-reverse-transcribed nucleic acids. Samples were analyzed using the same reagent batches and cycling conditions whenever possible to minimize inter-assay variability. The quantification cycle (Cq) values obtained from technical replicates were assessed for consistency. Replicates showing substantial variation were repeated according to predefined quality-control criteria. The mean Cq value of technically acceptable duplicate reactions was used for normalization and statistical analysis. The molecular workflow was conducted in accordance with the principles of the Minimum Information for Publication of Quantitative Real-Time PCR Experiments (MIQE) guidelines. The original manuscript supports the miScript II RT Kit, 500 ng of RNA, a 20-μL reaction volume, the miR-145-specific assay, Rotor-Gene Q, duplicate reactions, and the reported cycling protocol.

### 2.11. Normalization of miR-145 Expression

miR-145 expression was normalized against U6 small nuclear RNA (RNU6B), which was used as the endogenous reference RNA. U6 expression was assessed in all tissue samples, and its suitability as a normalization control was evaluated based on its consistency across the study groups and the technical reproducibility of its Cq values. Relative miR-145 expression was calculated using the comparative Cq method. The ΔCq value for each sample was calculated as follows:

$$\Delta \mathrm{Cq}\;={\;\mathrm{Cq}}_{\mathrm{miR}\text{-}145}\;-\;{\mathrm{Cq}}_{U6}$$

Relative expression was then calculated as

$$\mathrm{Relative}\;e\mathrm{xpression}={\;2}^{-\Delta \mathrm{Cq}}$$

For between-group statistical comparisons, ΔCq values were used because they are more appropriate for statistical analysis of qPCR data. Relative expression values were used for descriptive and graphical presentation. Where fold changes relative to the control group were presented, the 2^−ΔΔCq^ method was used, with the control group serving as the calibrator:

$$\Delta \Delta \mathrm{Cq}\;=\;{\Delta \mathrm{Cq}}_{\mathrm{sample}}\;-{\bar{\Delta \mathrm{Cq}}}_{\mathrm{control}}$$

$$\mathrm{Relative}\;\mathrm{fold}\;\mathrm{change}=2^{-\Delta \Delta \mathrm{Cq}}$$

The reference RNA and target assay were analyzed under the same experimental conditions. All normalization procedures, amplification conditions, technical replicates, and analytical calculations were documented to facilitate reproducibility.

### 2.12. Analysis of TGF-β Signaling and Vascular Smooth Muscle Cell Markers

To investigate the potential association between miR-145 expression, TGF-β signaling, and aortic wall remodeling, the expression of selected the TGF-β pathway components and vascular smooth muscle cell (VSMC) phenotype-related markers was evaluated at the mRNA and protein levels. The analyzed genes included TGFB1, TGFBR1, TGFBR2, SMAD2, SMAD3, and SMAD4. VSMC phenotype-related genes included ACTA2, TAGLN, CNN1, MYH11, and KLF4. Total RNA was extracted from aortic tissue using the RNA extraction procedure. For mRNA analysis, 1 μg of total RNA was reverse-transcribed into cDNA using a commercial reverse-transcription kit according to the manufacturer’s instructions. Quantitative real-time PCR was performed using gene-specific primers and a SYBR Green-based master mix. The expression of the target genes was normalized against GAPDH, which was used as the housekeeping gene in the protocol. Each sample was analyzed in duplicate, and the mean Cq value of technically acceptable reactions was used for subsequent calculations. Relative mRNA expression was calculated using the comparative Cq method, with the control group serving as the calibrator. The ΔCq values were used for statistical comparisons, whereas relative expression and fold-change values were used for graphical presentation. The mRNA analysis was complemented by the assessment of selected proteins involved in TGF-β signaling and VSMC phenotypic regulation, including TGF-β1, TGFBR1, TGFBR2, total SMAD2, phosphorylated SMAD2, total SMAD3, phosphorylated SMAD3, α-smooth muscle actin (α-SMA), TAGLN, MYH11, and KLF4, subject to tissue availability and the predefined experimental analysis plan. Verification required: The original manuscript identifies the target genes but lists GAPDH, ACTB, and RPLP0 as possible housekeeping genes without confirming the gene actually used.

### 2.13. Western Blot Analysis

For protein-level analysis, aortic tissue samples were homogenized on ice in radioimmunoprecipitation assay (RIPA) buffer supplemented with protease and phosphatase inhibitors. Aortic tissue samples were mechanically homogenized in ice-cold RIPA lysis buffer containing 25 mM Tris-HCl (pH 7.6), 150 mM NaCl, 1% NP-40, 1% sodium deoxycholate, and 0.1% SDS. Immediately before use, the buffer was supplemented with a protease inhibitor cocktail and a phosphatase inhibitor cocktail to minimize protein degradation and preserve phosphorylation status. Tissue homogenization was performed using a mechanical homogenizer under chilled conditions to minimize protein degradation and preserve phosphorylation status. The lysates were centrifuged at 10,000× *g* for 10 min at 4 °C and the resulting supernatants were collected for protein analysis. Total protein concentration was determined using the bicinchoninic acid (BCA) assay, according to the manufacturer’s instructions. Equal amounts of total protein, approximately 20–40 μg per sample, were separated by sodium dodecyl sulfate–polyacrylamide gel electrophoresis (SDS-PAGE). The separated proteins were electrotransferred onto polyvinylidene difluoride (PVDF) membranes. Membranes were blocked with 5% nonfat dry milk or bovine serum albumin, depending on the primary antibody requirements, for 30 min at room temperature. The membranes were incubated overnight at 4 °C with primary antibodies against TGF-β1 (Cell Signaling Technology [CST], Danvers, MA, USA; Cat. No. 3709), TGFBR1 (Proteintech, Rosemont, IL, USA; Cat. No. 55391-1-AP), TGFBR2 (CST; Cat. No. 41896), total SMAD2 (CST; Cat. No. 5339), phosphorylated SMAD2 at Ser465/467 (CST; Cat. No. 18338), total SMAD3 (CST; Cat. No. 9523), phosphorylated SMAD3 at Ser423/425 (CST; Cat. No. 9520), α-smooth muscle actin (α-SMA; CST; Cat. No. 19245), transgelin (TAGLN), myosin heavy chain 11 (MYH11), and Krüppel-like factor 4 (KLF4). All antibodies were used at the dilution recommended by the manufacturer or following optimization in preliminary experiments. After washing with Tris-buffered saline containing 0.1% Tween-20 (TBST), the membranes were incubated with the appropriate horseradish peroxidase-conjugated secondary antibody. Protein bands were detected using an enhanced chemiluminescence (ECL) detection system (Clarity Western ECL Substrate; Bio-Rad Laboratories, Hercules, CA, USA) and visualized using a ChemiDoc™ MP Imaging System (Bio-Rad Laboratories). Digital images were acquired using the Image Lab Software, version 6.1 (Bio-Rad Laboratories), and densitometric analysis was performed using ImageJ software (version 1.54; National Institutes of Health, Bethesda, MD, USA). The intensity of each target protein band was normalized to the corresponding loading-control band, and the normalized values were used for subsequent statistical analyses.

### 2.14. Assessment of the miR-145/TGF-β Remodeling Axis

The potential association between miR-145 expression, TGF-β signaling, VSMC phenotypic alterations, and aortic wall remodeling was evaluated by comparing molecular, histopathological, and clinical parameters between the AAA and control groups. The primary molecular comparisons included miR-145 expression in AAA and control aortic tissue; mRNA expression of TGFB1, TGFBR1, and TGFBR2; the expression of total and phosphorylated SMAD2 and SMAD3; and the expression of VSMC contractile and phenotypic-switching markers, including α-SMA, TAGLN, CNN1, MYH11, and KLF4. These molecular measurements were examined in relation to histopathological indicators of aortic wall remodeling, including medial degeneration, elastic-fiber disruption, collagen deposition, VSMC disorganization, inflammatory infiltration, and α-SMA-positive tissue area. Where appropriate, molecular findings were also compared with clinical characteristics, including maximal aortic diameter. Associations between miR-145 expression and molecular or histopathological parameters were evaluated using correlation analyses selected according to the distribution and measurement level of the variables. Spearman’s rank correlation coefficient was used for non-normally distributed continuous or ordinal variables. The analyses were conducted to identify relationships between miR-145 expression and the TGF-β pathway activity, VSMC phenotype-related markers, and structural remodeling indices. The observed associations were interpreted as correlations and were not considered evidence of a direct causal relationship between miR-145 downregulation, TGF-β signaling activation, or AAA progression. The molecular and histopathological findings were therefore integrated to describe a potential miR-145/TGF-β-associated remodeling pattern in AAA tissue.

### 2.15. qPCR Quality Control

Appropriate negative controls, including a no-template control and, where appropriate, a reverse-transcription-negative control, were used in all molecular assays. Technical replicates were tested for each sample. Amplification efficiency was determined via use of standard curves or by using an appropriate validated assay-performance procedure. Assays that displayed unfavorable amplification characteristics, nonspecific amplification or excess replication variance were repeated or were excluded if not conforming to preset quality-control criteria. Raw quantitative-cycle Cq values (sometimes reported as cycle threshold Ct) were retained for qualitative quality control purposes. No estimated Ct values were assigned to undetermined or technically unreliable amplification results. The molecular workflow followed the standard contemporary MIQE protocol procedures pertaining to sample handling, RNA quality, target identification, normalization, amplification performance, controls, and method of calculation of final results.

### 2.16. Statistical Analysis

Statistical analyses were performed using SPSS version 26 (IBM Corp., Armonk, NY, USA). For a better assessment of the distribution of continuous variables, they were tested with the Shapiro–Wilk test and by observing the histogram and the QQ-plot. Normally distributed data were reported as mean ± standard deviation, while non-normally distributed data were expressed as median (interquartile range, IQR). Categorical variables were reported as counts and percentiles. Student’s *t*-test for independent samples and the Mann–Whitney U test for non-normally distributed variables were used to compare the two groups. The chi-square test or Fisher’s exact test (for variables with small frequencies) was used for the comparison of two categorical variables. The Pearson correlation test was used for the assessment of the correlation between miR-145 expression level and clinical, biochemical, imaging, histological or molecular features; Spearman’s test was used for non-normally distributed features. A multiple regression analysis (linear or logistic, when appropriate) was applied to examine if the observed correlation between miR-145 and remodeling of the aorta remained after correction for clinically relevant variables, age, sex, hypertension, current/previous smoking, diabetes mellitus and other possible covariates selected a priori due to biological rationale and/or a potential role as confounders (the variable should not enter the model just based on univariable significance); if several molecular markers were examined, correction for multiple comparisons using the Benjamini–Hochberg method (controlling the false discovery rate) was performed. The results were reported with their associated 95% confidential interval (95% CI).

Statistical significance was set at a *p* value of less than 0.05, with all calculations two-tailed.

### 2.17. Ethical Considerations

The study protocol was reviewed and approved by the Ethics Committee of Tehran University of Medical Sciences on 28 March 2021 (approval number IR.TBZMED.VCR.REC.1401.066). The study was conducted in accordance with the Declaration of Helsinki and applicable institutional and national regulations governing research involving human participants and biological specimens. This was a 100% retrospective study based on previously collected and archived aortic tissue specimens and corresponding clinical records from patients treated between 2019 and 2025. No prospective recruitment, additional tissue sampling, or additional surgical intervention was performed for the purposes of this study. Previously archived specimens, including specimens collected before the date of ethics approval, were analyzed in accordance with the Ethics Committee’s determination permitting their retrospective use in anonymized form. The Ethics Committee determined that additional informed consent was not required for the retrospective analysis of these anonymized archived specimens and clinical data. All samples and corresponding clinical information were coded, de-identified, and analyzed anonymously.

## 3. Results

### 3.1. Study Population and Baseline Characteristics

In total, 648 participants were recruited in this study, including 324 patients with abdominal aortic aneurysm (AAA) and 324 controls. All patients and control individuals had adequate information for clinic and sufficient material for the histopathological and molecular examinations that were performed. Mean age was 67.4 ± 8.6 years in the overall study population. Patients with AAA were significantly older as compared to controls (69.1 ± 7.9 vs. 65.7 ± 8.9 years, *p* < 0.001). The majority of participants of both groups were males, but the percentage of males was significantly higher in AAA patients than in controls (78.1% vs. 65.7%, *p* < 0.001). The incidence of hypertension, smoking, diabetes mellitus and dyslipidemia was higher in AAA patients than in controls. Among the 324 AAA patients, 247 (76.2%) suffered from hypertension compared to 186 (57.4%) controls (*p* < 0.001). Current/former smoking was observed among 231 (71.3%) AAA patients and 164 (50.6%) controls (*p* < 0.001). Diabetes was observed among 92 (28.4%) AAA patients, compared to 64 (19.8%) controls (*p* = 0.013), and dyslipidemia was found among 181 (55.9%) AAA patients, compared to 132 (40.7%) control individuals (*p* < 0.001). The mean BMI of the recruited subjects was 27.4 ± 3.8 kg/m in the AAA group and 27.8 ± 4.1 kg/m in the control group (*p* = 0.214). There were no differences in BMI between cases and controls. Table 2 lists the summarized baseline characteristics of both groups.

### 3.2. Laboratory and Biochemical Characteristics

AAA patients compared to controls showed higher systemic inflammation. Median CRP was 4.8 (range 2.9–8.1) versus 2.6 (range 1.5–4.5) mg/L in AAA patients compared to controls, respectively, *p* < 0.001. In addition, there was a higher median leukocyte count of 8.1 (range 6.9–9.4) × 10^9^/L compared to the controls’ 6.9 (range 5.9–8.0) × 10^9^/L, *p* < 0.001. A high monocyte count was observed among AAA patients compared to controls: 0.71 (range 0.58–0.86) × 10^9^/L versus 0.54 (range 0.44–0.66) × 10^9^/L, *p* < 0.001. The HDL-C concentration was lower in AAA patients (43.2 ± 10.1 mg/dL) compared to the controls (48.6 ± 10.8 mg/dL), *p* < 0.001 (Table 3).

### 3.3. Histopathological Characteristics of the Aortic Wall

The histopathology confirmed prominent structural derangement in the specimens from patients with AAA as compared to control aortic tissue. H&E staining displayed thinning of the media, loss and misarrangement of the VSMCs and extracellular matrix, and increased infiltration by inflammatory cells. The semiquantitative medial degeneration score in the tissue was significantly higher in AAA as compared to control (AAA 2.8 ± 0.8; control 1.1 ± 0.5; *p* < 0.001).

Masson’s trichrome staining showed evidence of increased deposition of collagen and reorganization of extracellular matrix in the AAA tissue. Collagen-positive area in AAA tissue was 38.6 ± 9.7% as compared to control 26.9 ± 7.1% (*p* < 0.001). Elastic-fiber staining showed evidence of significant fragmentation of the elastic lamellae in AAA specimens; there was also gross derangement of the elastic lamellae in AAA tissue. Elastic-fiber derangement score in the AAA group was significantly higher than control (AAA 3.1 ± 0.7 vs. control 1.2 ± 0.5; *p* < 0.001). These findings confirmed widespread remodeling of extracellular matrix and loss of the normal medial structure in AAA (Table 4).

### 3.4. miR-145 Expression in AAA and Control Aortic Tissue

Total RNA yielding RNA molecules suitable for analysis by molecular assays was retrieved from all 648 tissue samples. The average RNA concentration found was 186.4 ± 72.3 ng/L with average A260/A280 and A260/A230 ratios of 1.96 and 2.08, respectively. Levels of mature miR-145 were substantially lower in AAA tissue when compared with control group tissue. The median normalized miR-145 expression was found to be 0.61 (IQR, 0.42–0.86) in AAA specimens and 1.00 (IQR, 0.72–1.34) in controls (*p* < 0.001). Individually calculated expressed values for miR-145 relative to control amounted to 0.61, approximately a 39% decrease in expression levels in AAA tissue relative to control tissue (see Figure 1 for illustration of individual values, medians and IQRs of data) (Table 5).

### 3.5. TGF-β Signaling in AAA Tissue

A significantly upregulated TGF-β signaling pathway was seen in the AAA tissue. The mean relative expression value of TGF-1 mRNA in AAA specimens compared to controls was 2.14 ± 0.81 vs. 1.00 ± 0.42 (*p* < 0.001). TGFBR1 was correspondingly expressed to a greater degree in the AAA specimens compared with control (1.68 ± 0.61 vs. 1.00 ± 0.37, *p* < 0.001). Similarly, the mean expression value of TGFBR2 in AAA specimens was greater than in control (1.54 ± 0.58 vs. 1.00 ± 0.35, *p* < 0.001). Corresponding to mRNA expression, a significant increase in the protein expression of TGF-1 was observed in AAA tissue (Figure 2). The relative difference in densitometric analysis values of protein expression was 1.9-fold greater compared with the controls (*p* < 0.001). The downstream SMAD signaling pathway was also activated; the ratio of p-SMAD2/SMAD2 was significantly higher in the AAA tissue compared with the controls (0.74 ± 0.19 vs. 0.42 ± 0.14, *p* < 0.001) while there was also a significantly higher ratio of p-SMAD3/SMAD3 in AAA samples versus control (0.68 ± 0.17 versus 0.39 ± 0.13, *p* < 0.001). This suggests the downstream SMAD pathway is active within the aneurysm samples, and in general a highly active TGF-/SMAD pathway is apparent within AAA tissue (Table 6).

### 3.6. Alterations in VSMC Phenotype

Contractile VSMC markers in AAA tissue: ACTA2 in AAA samples was expressed at 0.64 ± 0.25 relative units as compared with controls where it was 1.00 ± 0.31 (*p* < 0.001). TAGLN was similarly downregulated (0.59 ± 0.23 vs. 1.00±0.34, *p* < 0.001), as was MYH11 (0.55 ± 0.21 vs. 1.00 0.30, *p* < 0.001). In contrast, KLF4 expression was significantly elevated in AAA tissue (1.83 ± 0.69 vs. 1.00 ± 0.39, *p* < 0.001). An immunohistochemical study demonstrated decreased /c3bA staining and decreased SM22 staining in the medial layer of AAA tissue. Quantification of image analysis showed reduced %area which was %SMA-positive in tissue derived from AAA specimens compared to controls (42.1 ± 9.8% vs. 63.7 ± 10.4%, *p* < 0.001) (Table 7).

### 3.7. Association Between miR-145 and TGF-β Signaling

A significant inverse correlation was observed between miR-145 expression and TGF-β1 expression among the 648 participants (Spearman’s ρ = −0.48, *p* < 0.001). Similarly, miR-145 expression was inversely correlated with the p-SMAD2/SMAD2 ratio (ρ = −0.42, *p* < 0.001) and p-SMAD3/SMAD3 ratio (ρ = −0.39, *p* < 0.001). These associations were also observed when the AAA group was analyzed separately. Within AAA patients, miR-145 was inversely correlated with TGF-β1 expression (ρ = −0.44, *p* < 0.001), p-SMAD2/SMAD2 (ρ = −0.36, *p* < 0.001), and p-SMAD3/SMAD3 (ρ = −0.34, *p* < 0.001) (Table 8).

### 3.8. Association Between miR-145 and VSMC Phenotypic Markers

miR-145 expression demonstrated significant positive correlations with contractile VSMC markers. The strongest association was observed between miR-145 and MYH11 (ρ = 0.51, *p* < 0.001), followed by TAGLN (ρ = 0.47, *p* < 0.001) and ACTA2 (ρ = 0.44, *p* < 0.001). In contrast, miR-145 expression was inversely correlated with KLF4 expression (ρ = −0.43, *p* < 0.001). These findings demonstrated that lower miR-145 expression was associated with a less contractile and more phenotypically altered VSMC profile.

### 3.9. Association Between miR-145 and Aortic Wall Remodeling

Lower miR-145 expression was significantly associated with more severe histopathological remodeling. miR-145 expression showed an inverse correlation with the medial degeneration score (ρ = −0.46, *p* < 0.001) and elastic-fiber disruption score (ρ = −0.49, *p* < 0.001). Conversely, miR-145 expression was inversely correlated with collagen-positive area (ρ = −0.31, *p* < 0.001). Patients in the lowest tertile of miR-145 expression demonstrated significantly greater medial degeneration and elastic-fiber disruption than patients in the highest tertile (both *p* for trend < 0.001) (Table 9).

### 3.10. Relationship Between miR-145 and AAA Diameter

Within the AAA group, lower miR-145 expression was associated with a larger maximal aortic diameter. A significant inverse correlation was observed between miR-145 expression and maximal aortic diameter (Spearman’s ρ = −0.29, *p* < 0.001). Patients with an aortic diameter ≥60 mm had significantly lower miR-145 expression than those with an aortic diameter <60 mm (0.48 [IQR, 0.34–0.69] vs. 0.68 [IQR, 0.47–0.94], *p* = 0.002).

### 3.11. Associations with Clinical Characteristics

Within the AAA cohort, miR-145 expression was not significantly associated with age (ρ = −0.07, *p* = 0.19) or BMI (ρ = 0.04, *p* = 0.47). Patients with hypertension demonstrated modestly lower miR-145 expression than those without hypertension (0.58 vs. 0.69, *p* = 0.018). Similarly, current or former smokers had lower miR-145 expression than never-smokers (0.56 vs. 0.70, *p* = 0.006). No significant association was observed between miR-145 expression and diabetes mellitus (*p* = 0.21) or dyslipidemia (*p* = 0.09) (Table 10).

### 3.12. Multivariable Analysis

Multivariable linear regression was performed to determine whether miR-145 expression remained independently associated with aortic wall remodeling after adjustment for age, sex, hypertension, smoking status, diabetes mellitus, and maximal aortic diameter. After multivariable adjustment, miR-145 expression remained independently associated with the medial degeneration score (adjusted β = −0.31, 95% CI, −0.42 to −0.20; *p* < 0.001).

Similarly, miR-145 remained independently associated with the elastic-fiber disruption score (adjusted β = −0.34, 95% CI, −0.45 to −0.23; *p* < 0.001). In a separate model, lower miR-145 expression was independently associated with increased TGF-β1 expression (adjusted β = −0.37, 95% CI, −0.49 to −0.25; *p* < 0.001) (Table 11).

### 3.13. Integrated Molecular and Histopathological Findings

Taken together, the molecular and histopathological analyses demonstrated a consistent pattern characterized by reduced miR-145 expression, increased TGF-β1/TGFBR signaling, enhanced SMAD2/3 phosphorylation, reduced expression of contractile VSMC markers, increased KLF4 expression, and greater extracellular matrix remodeling in AAA tissue compared with control tissue. The magnitude of miR-145 reduction was significantly associated with the extent of TGF-β/SMAD pathway activation and the severity of aortic wall structural abnormalities. The associations remained statistically significant after adjustment for major demographic and cardiovascular risk factors.

## 4. Discussion

In the current study, we investigated the association between miR-145 expression, TGF-β signaling, vascular smooth muscle cell (VSMC) phenotype, and structural remodeling of the aortic wall in patients with abdominal aortic aneurysm (AAA). The main findings were that miR-145 expression was lower in AAA tissue, whereas TGF-β1/TGFBR signaling and SMAD2/3 activation were increased. Reduced miR-145 expression was also associated with decreased expression of contractile VSMC markers, increased KLF4 expression, greater medial degeneration, elastic-fiber disruption, and a larger maximal aortic diameter. The associations between VSMC-related markers, KLF4 expression, and aortic wall remodeling remained significant after adjustment for relevant clinical variables. Overall, these findings suggest that dysregulation of the miR-145–TGF-β/VSMC axis is associated with molecular and structural remodeling of the aneurysmal aortic wall. The observed findings included altered miR-145 expression, changes in TGF-β-related signaling markers, reduced expression of contractile VSMC markers, and histopathological features of aortic wall remodeling. These findings indicate that miR-145 expression is associated with molecular and structural characteristics of AAA; however, they do not establish a direct or functional miR-145–TGF-β/VSMC signaling axis.

A decrease in the expression of miR-145 in AAA tissue is biologically rational and supported by existing experimental evidence that points to miR-145 involvement in vascular smooth muscle (VSMC) homeostasis [24]. MiR-145 is among the most abundantly expressed miRNAs in terminally differentiated VSMCs and plays a role in the preservation of the contractile VSMC phenotype by regulating the transcriptional programs that control VSMC differentiation, contractility, proliferation, and migration [8,9,10]. Prior studies have documented an association of the absence of miR-145 with VSMC phenotypic switching, whereas its reintroduction resulted in partial maintenance of the differentiated phenotype [25]. Indeed, AAA transcriptomic studies also indicate a possible role of miR-145 in AAA pathogenic mechanisms [26]. One genome-wide screening showed downregulation of miR-145 expression and suggested interactions with inflammatory genes as well as genes involved in vascular biology, such as RAC2 and SELL, in AAA tissue [27]. Furthermore, other initial miRNA-screening studies revealed human AAA tissue displaying unique miRNA profiles when compared with non-aneurysmal control aorta [28], suggesting the implication of post-transcriptional mechanisms in the pathogenesis of the aortic wall remodeling process. Recently, another in-depth analysis that focused on human AAA confirmed the widespread up/downregulation of various miRNAs in both vascular tissues and blood, albeit with divergent individual miRNA expression levels in different studies [29].

Thus, by exploring the regulation of miR-145, in the context of aortic wall remodeling, rather than simply noting differences in miRNA expression profile, our findings should build upon current knowledge. The correlations between miR-145 reduction with medial degeneration, degradation of elastic fibers, and increased aneurysm diameter indicated that miR-145 levels can also indicate the biological condition of the aneurysmal wall, although caution needs to be applied in interpretation. Variations in miRNA levels can occur due to differences in cell type present, disease stage and location, as well as influence of drugs and inflammation. Therefore, it is not automatic that the decrease in miR-145 in the aorta indicates that loss of miR-145 expression caused AAA; instead, current data shows that dysregulation of miR-145 correlates to the remodeling phenotype.

An important finding of the present study was the association between reduced miR-145 expression and VSMC phenotypic alterations. In AAA tissue, lower expression of ACTA2, TAGLN, CNN1, and MYH11 and higher KLF4 expression were associated with the observed molecular and structural remodeling pattern. These findings are consistent with the established role of VSMC phenotypic plasticity in aortic aneurysmal disease. VSMCs may transition from a differentiated contractile phenotype toward alternative phenotypes, including synthetic, proliferative, migratory, and inflammation-associated states, which may contribute to extracellular matrix degradation and weakening of the aortic wall [29,30,31]. Previous studies of human AAA tissue have also reported reduced expression of contractile markers, including α-SMA and SM22, together with impaired cellular organization and differentiation within the aortic wall [32]. Our findings are consistent with these previously described alterations in VSMC phenotype. The association between altered miR-145 expression and VSMC phenotypic markers may be relevant to the vascular remodeling observed in AAA. The reduced expression of contractile markers and the altered expression of markers related to VSMC phenotype are consistent with the involvement of VSMC phenotypic changes in aneurysmal disease. Nevertheless, the cross-sectional observational design does not allow us to determine whether changes in miR-145 expression precede VSMC phenotypic alterations or whether they arise as a consequence of the disease process. Furthermore, because miR-145 was not experimentally manipulated in the present study, a direct regulatory effect on VSMC phenotype cannot be inferred.

Perhaps the interaction with KLF4 is of greatest interest. Qiu et al. [13] reported that miR-145 was indeed markedly downregulated in both human aortic dissection tissues and experimental models. Mechanical strain induced KLF4 expression and enhanced VSMC phenotypic transition.

Crucially, enforced expression of miR-145 could counteract the mechanically induced increment in KLF4, and inhibit VSMC phenotypic transition. That we found an inverse correlation between miR-145 and KLF4 is therefore consistent with an existing mechanism whereby suppression of miR-145 may contribute to VSMC phenotypical remodeling. Similarly, a study in atherosclerotic aorta demonstrated that decreased level of miR-145 correlated with increased VSMC proliferation and inhibition of contractile phenotypes and that partially restored levels of miR-145 partially reversed this alteration [33]. Although atherosclerosis and AAA are different diseases, they have significant similarities in VSMC phenotypic plasticity and extracellular matrix remodeling, so this would be reasonable.

The primary aim of the current study was to investigate whether miR-145 dysregulation was associated with TGF-β signaling in the aneurysmal aortic wall. The findings demonstrated increased expression of TGF-β1, TGFBR1, and TGFBR2, together with increased p-SMAD2/SMAD2 and p-SMAD3/SMAD3 ratios, in AAA tissue compared with control tissue. Conversely, lower miR-145 expression was inversely associated with TGF-β1 expression and indicators of SMAD2/3 activation in AAA tissue. TGF-β signaling is relevant to vascular biology because it regulates VSMC differentiation, extracellular matrix synthesis, cell proliferation, apoptosis, and inflammation [34]. The role of TGF-β signaling in aneurysmal disease is complex and may depend on the anatomical site, disease stage, cellular context, and balance between canonical and non-canonical signaling pathways. Although increased TGF-β signaling was observed in AAA tissue in the present study, these findings should not be interpreted as evidence that increased TGF-β activity necessarily causes aneurysm formation. The inverse association between miR-145 expression and the TGF-β pathway-related measurements suggests that these regulatory systems may be related during aneurysmal wall remodeling. However, further mechanistic studies are needed to determine the direction and biological significance of this relationship.

Furthermore, the role of TGF- signaling in aneurysm development is complicated and appears site- and stage-specific and dependent on individual cell types and the interplay between both canonical and non-canonical pathways. Existing human data support the increased levels of TGF-1 expression in AAA tissue [35]. In a single earlier work performed in human AAA tissue both in aneurysmal neck tissue and in the dilative region, higher expression levels were seen in conjunction with low SMCS [36]. Similarly, disabling of TGF- signaling exclusively within SMCs can alter experimental AAA development, where disabling TGFBR2 limits medial SMC loss and extracellular matrix degradation and reduces MPO and macrophage infiltration in an experimental AAA model [37]. However, high levels of activated TGF- signaling within AAA tissues can be interpreted in a number of ways. Previous studies have already suggested protective effects of canonical TGF- signaling pathway against AAA formation by reducing inflammation, EC degradation and SMC apoptosis [38]. Hence, it could be explained as a mechanism compensating for damaged vascular wall. Accordingly, our findings should not be interpreted as demonstrating that increased TGF-β signaling necessarily causes AAA. Rather, the observed inverse relationship between miR-145 and TGF-β pathway activity suggests that these two regulatory systems are closely associated during aneurysmal wall remodeling.

Another fascinating feature of the findings that we report here is their lack of absolute consistency with prior publications. One prior report from the laboratory of Pei et al. [39], looking at aneurysmal tissue of the ascending aorta, found elevated, not decreased, miR-145 expression in the aneurysmal sample compared with that obtained from control samples. This work in 10 patients with ascending aortic aneurysm and 10 subjects without aneurysm reported elevated levels of miR-145, OPN, and collagen type III in the ascending aortic tissue from patients versus normal subjects. Moreover, their in vitro work with vascular cells supported the idea that miR-145 stimulation caused increased expression of OPN and collagen type III via action through the TFG-beta pathway [40]. At first glance, these findings may appear inconsistent with the reduced miR-145 expression observed in the present study on abdominal aortic aneurysm. However, abdominal and thoracic aortic aneurysms are distinct disease entities and may involve different biological processes. TGF-β signaling may vary according to the anatomical location of the disease, genetic background, local hemodynamic conditions, disease stage, and cellular composition of the affected tissue. Therefore, findings from ascending aortic aneurysm should not necessarily be extrapolated directly to AAA. The observed differences may reflect disease- and location-specific regulatory mechanisms. Further comparative studies are required to clarify the context-dependent role of miR-145 in different forms of aortic disease. This is why some of reviews highlight these dichotomies depending on the particular circumstance and situation. In essence and as noted, the experiments from Pei et al. [39] involved the thoracic aorta while this current study examines an abdominal process and the disparate location leads to different underlying biology. The fact that our data is more in keeping with findings by another research team in an experimental model of abdominal aneurysm induced by AngII (where increased expression of miR-145 was demonstrated to limit aneurysmal disease incidence, diameter and inflammatory factors like collagen 3 loss and Mmp2 activation) strengthens the premise that miR-145 does exert a function in abdominal aneurysm development that is disease-setting-specific and even location-specific as we previously mentioned [41].

The relationship between reduced miR-145 expression and increased medial degeneration and elastic-fiber disruption is biologically relevant. The maintenance of a structurally intact aortic media depends on coordinated interactions among elastin, collagen, and VSMCs [42]. During AAA, disruption of these components may reduce the mechanical strength of the aortic wall and contribute to progressive remodeling. In the present study, lower miR-145 expression was associated with medial degeneration, elastic-fiber disruption, and increased aortic diameter. These findings are consistent with previous experimental evidence suggesting that miR-145 may influence VSMC phenotype and extracellular matrix homeostasis. Nevertheless, the observed associations do not establish that reduced miR-145 expression directly causes extracellular matrix degradation. Reduced miR-145 expression may precede remodeling, result from the altered cellular and inflammatory environment, or reflect both processes. Longitudinal and mechanistic studies are required to clarify the temporal and causal relationships.

In addition, the observed correlation of miR-145 and MYH11, TAGLN, and ACTA2 indicates that miR-145 may be linked to the maintenance of contractility in the VSMC and hence conservation of the extracellular matrix. Dedifferentiated VSMCs would therefore indirectly affect extracellular matrix degradation by decreasing the medial layer’s potential for maintenance of its constituent elements. As this study is observational, however, the reduced miR-145 level can either precede or be a result of extracellular matrix breakdown caused by the disturbed cellular and inflammatory context of the aneurysm, and future longitudinal and mechanistic study is required.

The inverse relationship between miR-145 expression and maximal aortic diameter represents another relevant finding. Participants with larger aneurysms had lower miR-145 expression, and miR-145 remained associated with remodeling-related parameters after adjustment for selected clinical factors. This association should be interpreted cautiously because aneurysm growth is influenced by disease duration, inflammation, extracellular matrix degradation, mechanical stress, and cardiovascular risk factors. The cross-sectional relationship observed in the present study does not establish that reduced miR-145 expression precedes aneurysm enlargement or predicts subsequent aneurysm growth or rupture. Rather, lower miR-145 expression may be associated with a more advanced molecular or structural remodeling phenotype. Prospective studies measuring miR-145 expression before and during aneurysm progression are needed to determine its temporal relationship with aortic enlargement and its potential clinical relevance.

This is in agreement with the fact that, depending on the stage of the disease, different molecular changes may occur within the wall of the aneurysmal sac. Nevertheless, as with the cross-sectional relation of miR-145 with aneurysm diameter, this must not be understood to indicate that miR-145 independently precedes the future aneurysm dilatation or rupture. Aneurysm growth depends on the disease time course, inflammation, breakdown of extracellular matrix, stresses in the aortic wall and various cardiovascular risk factors. Consequently, low miR-145 expression implies a more advanced morphological phenotype remodeling process and not that miR-145 precedes the future aneurysm diameter increase and potential rupture. A prospective investigation measuring miR-145 at baseline and after aneurysmal changes should be carried out before it can be introduced in clinic. Taken together, the findings support a conceptual model in which miR-145 participates in the regulatory network connecting VSMC phenotype with extracellular matrix remodeling in AAA.

Physiologically, miR-145 is constitutively highly expressed in differentiated VSMCs and maintains the contractile phenotype [39,40]. Downregulation of miR-145 within the aneurysmal microenvironment may promote the phenotypic plasticity of VSMCs. In this situation VSMCs may show reduced expression of ACTA2, TAGLN, and MYH11 and up-regulation of KLF4 [43]. In parallel, alterations in TGF- signaling may modulate VSMC activity and ECM turnover [44]. The present findings demonstrate associations between reduced miR-145 expression, TGF-β/SMAD pathway-related markers, VSMC phenotypic alterations, and aortic wall remodeling in AAA tissue. Although these relationships are biologically plausible and consistent with previously reported roles of miR-145 in vascular smooth muscle cell regulation, they should not be interpreted as evidence of a direct or functional miR-145–TGF-β/VSMC signaling axis. The observational design does not establish the directionality of these relationships or determine whether miR-145 dysregulation directly contributes to the TGF-β pathway activation or VSMC phenotypic switching.

The subsequent phenotype would be conducive to disruption of the medial architecture, loss and fragmentation of elastic fibers, mal-adaptation of collagen remodeling, and progressive aortic dilation. This is, of course, a model based on associations identified within aneurysms and not a direct causal pathway. It is almost certain that the interaction of miR-145 and TGF- is dynamic and bi-directional, and may additionally involve TGF--dependent signaling modulating miR-143/145 and transfer of miR-143/145 by both miR-200 and TGF-beta [45]. It is unlikely to be a simple, one-way process of miR-145 regulation by TGF- or vice versa.

The current observations do have several potential translational ramifications. miRNAs have been considered suitable for potential biomarker development due to their relative stability and possibility for assay in circulating blood. Although the change in tissue expression and change in circulating levels are not always parallel, the circulating expression of miRNAs and their respective impact on the biological properties of the local aorta-wall cells are still unclear. The only previous systematic review on circulating miRNA in aneurysm specifically listed miR-145, among others; it showed it to be a repeated target, and underlined the necessity of standardized selection criteria, material obtained and methodology for miRNA analysis. Thus, while the present data offer evidence towards the role of miR-145 as a biological marker for the remodeling associated with AAA-induced wall cells, they may not determine its diagnostic nor prognostic value in the clinic.

Considering a potential therapeutic option, the exogenous expression of miR-145 has led to some potentially beneficial effects in models of AAA. For the AngII model of aneurysm formation, an overexpression of miR-145 was associated with reduction in aneurysmal incidence and attenuation in elastin degradation. For other disease models of vessel pathology, the exogenous restoration of miR-145 to affect VSMCs has already been documented to a certain extent. This opens up the perspective for the treatment of aneurysm disease on the basis of such an approach affecting the remodeling process at the level of the vessel wall in the future. Nevertheless, this prospect remains speculative and in contradiction to itself in various forms of aneurysms.

Peripheral arterial disease was also considered as an indicator of systemic atherosclerotic burden. The coexistence of PAD and AAA may reflect shared vascular risk factors and overlapping mechanisms of vascular wall injury, although AAA is a complex degenerative process that cannot be considered simply a manifestation of atherosclerosis. Therefore, the presence of systemic arterial disease should be taken into account when interpreting molecular alterations associated with aortic wall degeneration and remodeling. In the present study, PAD was included among the clinical characteristics of the study population to provide a more comprehensive assessment of concomitant vascular disease.

The primary strength of the current work is the combination, in a relatively large cohort, of clinicopathologic variables, histopathology, miR-145 expression, TGF signaling, VSMC phenotype and aortic wall remodeling. Because we performed individual molecular measurements and correlated them with other variables, the approach possesses higher biologic resolution than studies that compared large groups. Several limitations should be considered. First, the observational design allows the identification of associations but does not establish causality or temporal relationships among miR-145 expression, TGF-β/SMAD signaling-related markers, VSMC phenotypic markers, and aortic wall remodeling. Second, although the observed molecular patterns are biologically consistent with a potential relationship between miR-145 and vascular remodeling, we did not perform miR-145 overexpression or inhibition experiments, direct target-validation assays, or pathway-specific rescue experiments. Therefore, a functional miR-145–TGF-β/VSMC signaling axis cannot be confirmed. Further mechanistic studies are needed to determine the directionality, molecular targets, and functional consequences of these associations. The observational design does not permit conclusions about causation. Aortic tissue collected at surgery represents advanced AAA and may not capture events occurring early in the process of development. Tissue measurements of miRNA do not permit assignment of miR-145 to specific cell types, such as those of the endothelium, fibroblast, smooth muscle, inflammatory or infiltrating immune cell populations. Surgery in patients introduces variability concerning age, the presence of vascular risk factors, medications and site of collection of the aorta, which ought to be taken into consideration in a multivariable analysis. The correlation between the presence of miR-145 and TGF/SMAD signaling is biologically plausible but does not in itself suggest physical targeting of the TGF signaling pathway. Functional biologic work such as luciferase assay and gain- and loss-of-function experiments in the different cell types should be carried out prove the direct association between these two molecular mechanisms. Further work in independent cohorts should be used to confirm the present results, and ideally biologic or circulatory miR-145 should be correlated with subsequent expansion. Importantly, this study did not include miR-145 overexpression or inhibition experiments, direct target-validation assays, or pathway-specific rescue experiments. Therefore, the observed associations cannot establish direct molecular targeting, pathway directionality, or causality. In addition, tissue-level measurements do not establish cell-type-specific mechanisms. Future studies incorporating gain- and loss-of-function approaches, direct target-validation assays, and cell-specific experimental models are needed to determine whether miR-145 directly regulates TGF-β/SMAD signaling and VSMC phenotypic changes in AAA.

## 5. Conclusions

Overall, this study identified an association between reduced miR-145 expression and molecular and structural remodeling of the aneurysmal aortic wall. Lower miR-145 expression was associated with increased TGF-β/SMAD pathway-related activity, reduced expression of contractile VSMC markers, increased KLF4 expression, extracellular matrix remodeling, elastic-fiber disruption, and greater maximal aortic diameter. These findings support a potential relationship between the miR-145–TGF-β/VSMC axis and aortic wall remodeling in AAA. However, the observational design does not establish that miR-145 dysregulation directly causes aneurysm formation or progression. Further longitudinal and mechanistic studies are required to clarify the biological role of miR-145, determine the direction of the observed associations, and evaluate its potential clinical significance.

## Figures and Tables

**Figure 1:**
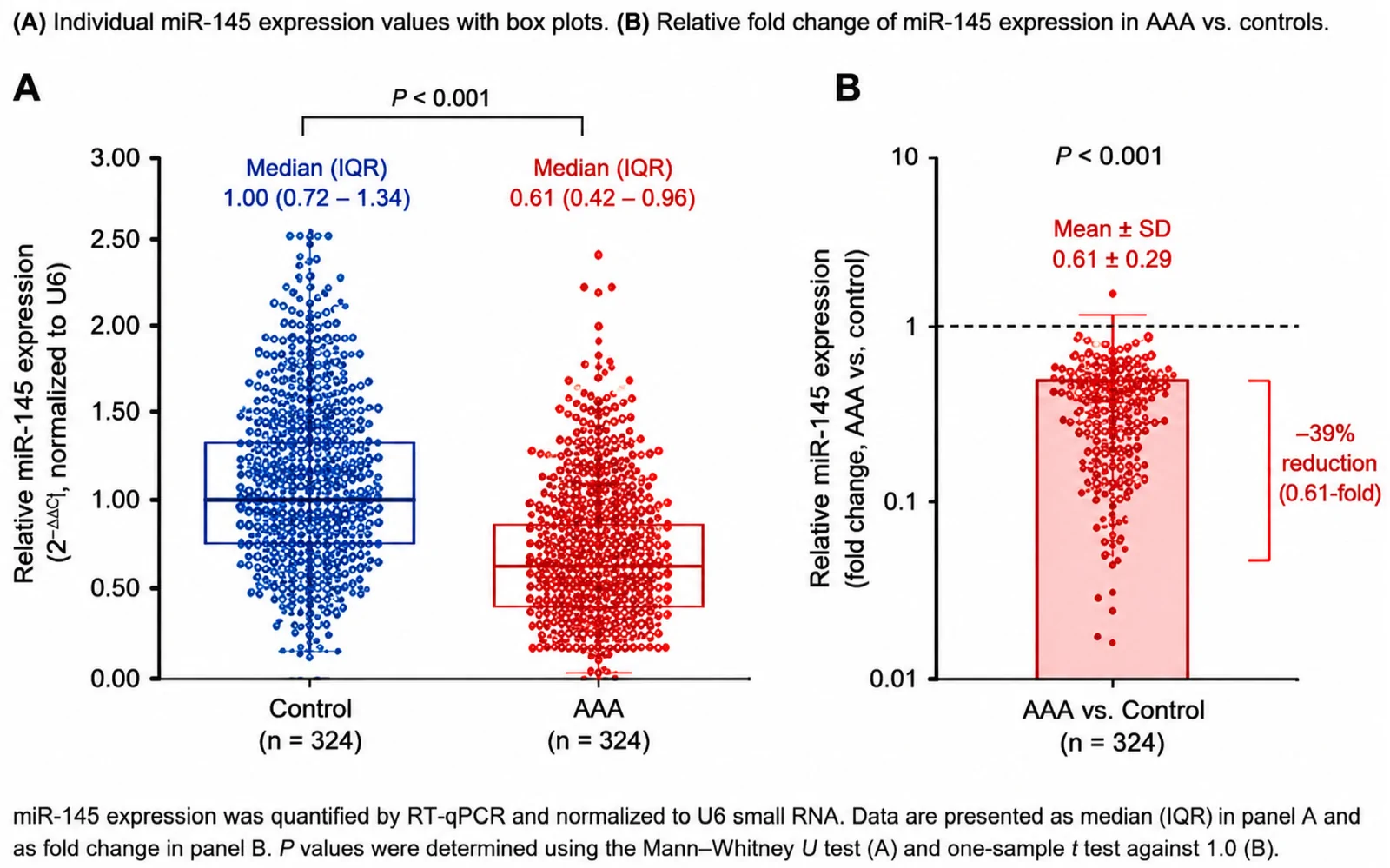
Differential expression of miR-145 in aortic tissue from patients with AAA and controls. Individual miR-145 expression values are shown for all participants. Horizontal lines represent the median and interquartile range. miR-145 expression was significantly lower in AAA tissue than in control aortic tissue (median, 0.61 [IQR, 0.42–0.86] vs. 1.00 [IQR, 0.72–1.34], *p* < 0.001).

**Figure 2:**
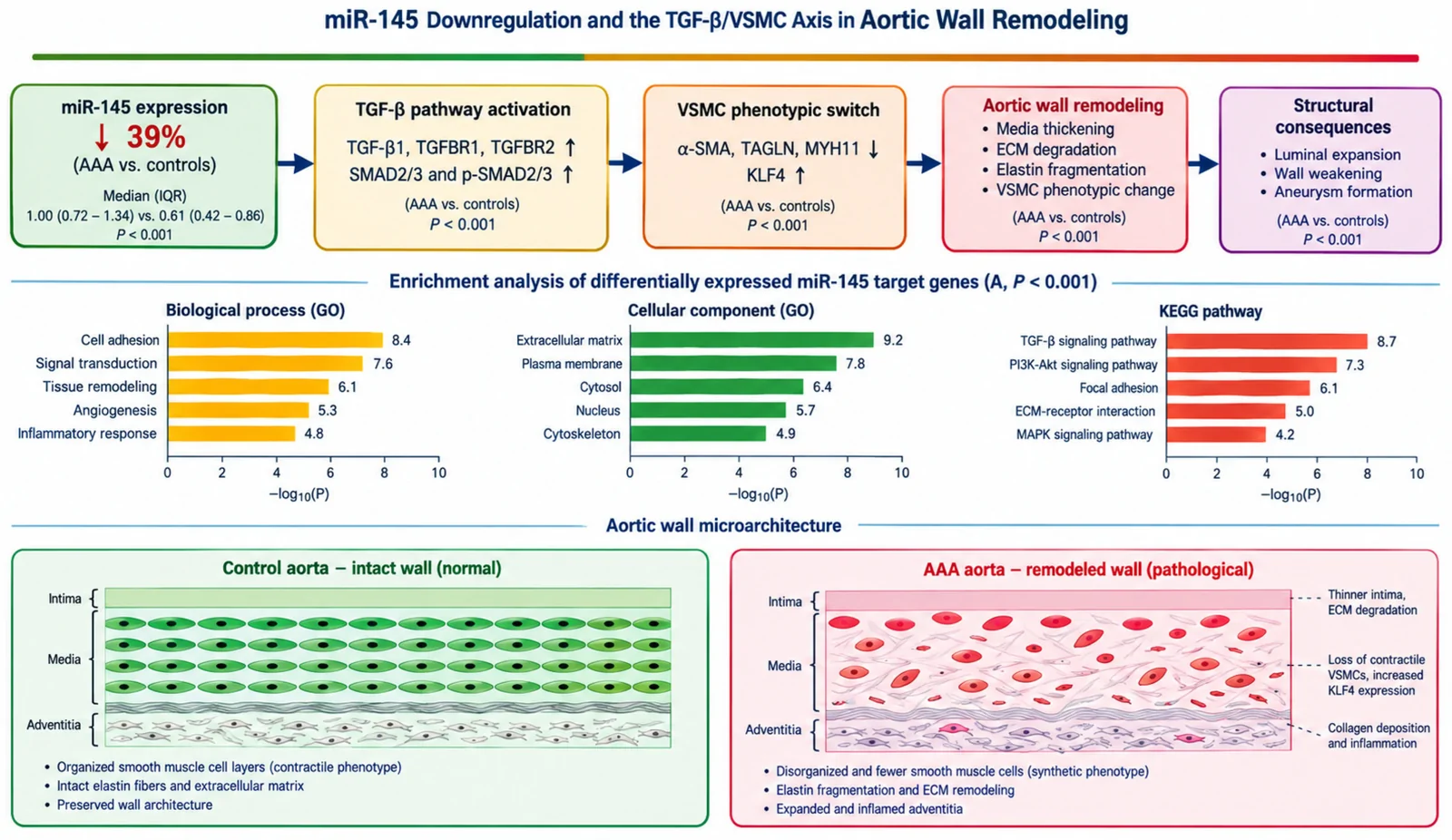
Correlation between miR-145 and TGF-β signaling (miR-145 downregulation is associated with TGF-β/SMAD activation, VSMC phenotypic switching, and structural remodeling of the aortic wall in abdominal aortic aneurysm (AAA)).

**Table 1:** Primary antibodies used for immunohistochemical assessment of VSMC and TGF-β signaling markers.

Target Antigen	Manufacturer	Catalog No.	Clone/Host	Simulated Dilution
α-SMA (ACTA2)	Cell Signaling Technology (CST), Danvers, MA, USA	19245	D4K9N; rabbit monoclonal antibody	1:200
SM22α/TAGLN	Abcam, Cambridge, UK	ab14106	Host and clone require verification from the product datasheet	1:200
Calponin (CNN1)	Abcam, Cambridge, UK	ab46794	Host and clone require verification from the product datasheet	1:200
TGF-β1	Cell Signaling Technology, Danvers, MA, USA	3709	56E4; rabbit monoclonal antibody	1:200
TGF-β receptor II (TGFBR2)	Cell Signaling Technology, Danvers, MA, USA	41896	E5M6F; rabbit monoclonal antibody	1:200
Total SMAD2	Cell Signaling Technology, Danvers, MA, USA	5339	D43B4; rabbit monoclonal antibody	1:200
Phosphorylated SMAD2 (Ser465/467)	Cell Signaling Technology, Danvers, MA, USA	18338	E8F3R; rabbit monoclonal antibody	1:200
Total SMAD3	Cell Signaling Technology, Danvers, MA, USA	9523	Clone and host require verification; rabbit antibody	1:200
Phosphorylated SMAD3 (Ser423/425)	Cell Signaling Technology, Danvers, MA, USA	9520	Clone and host require verification; rabbit antibody	1:200

**Table 2:** Demographic and clinical characteristics of the study population.

Variable	AAA Group (n = 324)	Control Group (n = 324)	*p* Value
Age, years	69.1 ± 7.9	65.7 ± 8.9	<0.001
Male sex, n (%)	253 (78.1)	213 (65.7)	<0.001
BMI, kg/m^2^	27.4 ± 3.8	27.8 ± 4.1	0.214
Hypertension, n (%)	247 (76.2)	186 (57.4)	<0.001
Diabetes mellitus, n (%)	92 (28.4)	64 (19.8)	0.013
Dyslipidemia, n (%)	181 (55.9)	132 (40.7)	<0.001
Current/former smoking, n (%)	231 (71.3)	164 (50.6)	<0.001
Coronary artery disease, n (%)	88 (27.2)	53 (16.4)	0.001
Chronic kidney disease, n (%)	31 (9.6)	19 (5.9)	0.087
Peripheral arterial disease, n (%)	72 (22.2)	24 (7.4)	<0.001
Statin use, n (%)	126 (38.9)	91 (28.1)	0.005
Antihypertensive therapy, n (%)	231 (71.3)	171 (52.8)	<0.001
Antiplatelet therapy, n (%)	101 (31.2)	72 (22.2)	0.009

Data are presented as mean ± standard deviation or number (percentage). *p* values were obtained using the independent-samples *t*-test or χ^2^/Fisher’s exact test, as appropriate. AAA: abdominal aortic aneurysm; BMI: body mass index.

**Table 3:** Laboratory and biochemical characteristics of the study groups.

Variable	AAA Group (n = 324)	Control Group (n = 324)	*p* Value
Leukocyte count, ×10^9^/L	8.1 (6.9–9.4)	6.9 (5.9–8.0)	<0.001
Neutrophil count, ×10^9^/L	5.8 (4.8–6.9)	4.6 (3.8–5.6)	<0.001
Monocyte count, ×10^9^/L	0.71 (0.58–0.86)	0.54 (0.44–0.66)	<0.001
Lymphocyte count, ×10^9^/L	1.72 (1.40–2.06)	1.88 (1.55–2.22)	0.006
CRP, mg/L	4.8 (2.9–8.1)	2.6 (1.5–4.5)	<0.001
Total cholesterol, mg/dL	192.4 ± 38.1	181.6 ± 34.7	<0.001
LDL-C, mg/dL	118.7 ± 31.2	108.5 ± 28.9	<0.001
HDL-C, mg/dL	43.2 ± 10.1	48.6 ± 10.8	<0.001
Triglycerides, mg/dL	154.6 (118.0–202.0)	136.5 (101.0–178.0)	0.002
Glucose, mg/dL	108.2 (94.0–129.0)	101.4 (89.0–116.0)	0.003
Creatinine, mg/dL	1.02 ± 0.28	0.96 ± 0.23	0.006

Data are presented as mean ± standard deviation or median (interquartile range). AAA: abdominal aortic aneurysm; CRP: C-reactive protein; HDL: high-density lipoprotein; LDL: low-density lipoprotein.

**Table 4:** Histopathological characteristics of aortic tissue.

Histopathological Parameter	AAA (n = 324)	Control (n = 324)	*p* Value
Medial degeneration score	2.8 ± 0.8	1.1 ± 0.5	<0.001
Elastic-fiber disruption score	3.1 ± 0.7	1.2 ± 0.5	<0.001
Collagen-positive area, %	38.6 ± 9.7	26.9 ± 7.1	<0.001
VSMC disorganization score	2.7 ± 0.8	1.0 ± 0.4	<0.001
Inflammatory infiltration score	2.2 ± 0.9	0.9 ± 0.4	<0.001
α-SMA-positive area, %	42.1 ± 9.8	63.7 ± 10.4	<0.001

Higher scores indicate more severe pathological alterations. Data are presented as mean ± standard deviation or median (interquartile range). AAA: abdominal aortic aneurysm; VSMC: vascular smooth muscle cell.

**Table 5:** miR-145 expression in AAA and control aortic tissue.

Molecular Parameter	AAA (n = 324)	Control (n = 324)	*p* Value
RNA concentration, ng/μL	184.7 ± 69.8	188.1 ± 74.2	0.563
A260/A280 ratio	1.95 ± 0.08	1.96 ± 0.07	0.118
A260/A230 ratio	2.06 ± 0.19	2.09 ± 0.18	0.071
miR-145 relative expression	0.61 (0.42–0.86)	1.00 (0.72–1.34)	<0.001
miR-145 fold change	0.61 ± 0.29	1.00 ± 0.42	<0.001

Data are presented as mean ± standard deviation or median (interquartile range). AAA: abdominal aortic aneurysm.

**Table 6:** Expression of TGF-β signaling-related genes and proteins.

Marker	AAA	Control	*p* Value
TGFB1 mRNA	2.14 ± 0.81	1.00 ± 0.42	<0.001
TGFBR1 mRNA	1.68 ± 0.61	1.00 ± 0.37	<0.001
TGFBR2 mRNA	1.54 ± 0.58	1.00 ± 0.35	<0.001
TGF-β1 protein	1.90 ± 0.61	1.00 ± 0.34	<0.001
p-SMAD2/SMAD2	0.74 ± 0.19	0.42 ± 0.14	<0.001
p-SMAD3/SMAD3	0.68 ± 0.17	0.39 ± 0.13	<0.001

Data are presented as mean ± standard deviation or median (interquartile range).

**Table 7:** Vascular smooth muscle cell phenotype-related molecular markers.

Marker	AAA	Control	*p* Value
ACTA2	0.64 ± 0.25	1.00 ± 0.31	<0.001
TAGLN	0.59 ± 0.23	1.00 ± 0.34	<0.001
CNN1	0.63 ± 0.24	1.00 ± 0.30	<0.001
MYH11	0.55 ± 0.21	1.00 ± 0.30	<0.001
KLF4	1.83 ± 0.69	1.00 ± 0.39	<0.001
α-SMA immunohistochemical staining, %	42.1 ± 9.8	63.7 ± 10.4	<0.001
SM22α-positive area, %	38.7 ± 8.9	59.8 ± 10.1	<0.001

Data are presented as mean ± standard deviation or median (interquartile range). Gene-expression values are presented as relative expression normalized to validated housekeeping genes. AAA: abdominal aortic aneurysm.

**Table 8:** Correlations between miR-145 and molecular markers of TGF-β signaling and VSMC phenotype.

Variable Correlated with miR-145	Spearman’s ρ	*p* Value
TGF-β1	−0.48	<0.001
TGFBR1	−0.36	<0.001
TGFBR2	−0.32	<0.001
p-SMAD2/SMAD2	−0.42	<0.001
p-SMAD3/SMAD3	−0.39	<0.001
ACTA2	+0.44	<0.001
TAGLN	+0.47	<0.001
CNN1	+0.41	<0.001
MYH11	+0.51	<0.001
KLF4	−0.43	<0.001

**Table 9:** Correlations Between miR-145 expression and aortic wall remodeling.

Remodeling Parameter	Spearman’s ρ	*p* Value
Medial degeneration score	−0.46	<0.001
Elastic-fiber disruption score	−0.49	<0.001
Collagen-positive area	−0.31	<0.001
VSMC disorganization score	−0.43	<0.001
Inflammatory infiltration score	−0.28	<0.001
Maximal aortic diameter	−0.29	<0.001

VSMC: vascular smooth muscle cell.

**Table 10:** Association between miR-145 expression and clinical characteristics in patients with AAA.

Variable	miR-145 Expression	*p* Value
Age, Spearman’s ρ	−0.07	0.190
BMI, Spearman’s ρ	+0.04	0.470
Hypertension	0.58 vs. 0.69	0.018
Diabetes mellitus	0.59 vs. 0.63	0.210
Dyslipidemia	0.58 vs. 0.64	0.090
Current/former smoking	0.56 vs. 0.70	0.006
Statin use	0.60 vs. 0.62	0.640
Maximal aortic diameter, Spearman’s ρ	−0.29	<0.001

BMI: body mass index.

**Table 11:** Multivariable regression analysis of factors associated with aortic wall remodeling.

Dependent Variable	Independent Variable	Adjusted β	95% CI	*p* Value
Medial degeneration score	miR-145	−0.31	−0.42 to −0.20	<0.001
	Age	+0.14	0.05 to 0.23	0.002
	Male sex	+0.08	−0.01 to 0.17	0.084
	Hypertension	+0.12	0.03 to 0.21	0.009
	Smoking	+0.15	0.06 to 0.24	0.001
	Diabetes mellitus	+0.06	−0.03 to 0.15	0.192
	Aortic diameter	+0.27	0.18 to 0.36	<0.001
Elastic-fiber disruption score	miR-145	−0.34	−0.45 to −0.23	<0.001
	Age	+0.11	0.02 to 0.20	0.018
	Hypertension	+0.10	0.01 to 0.19	0.031
	Smoking	+0.17	0.08 to 0.26	<0.001
	Aortic diameter	+0.30	0.21 to 0.39	<0.001
TGF-β1 expression	miR-145	−0.37	−0.49 to −0.25	<0.001
	Age	+0.09	0.00 to 0.18	0.046
	Hypertension	+0.11	0.02 to 0.20	0.017
	Smoking	+0.13	0.04 to 0.22	0.005

CI, confidence interval. Models were adjusted for age, sex, hypertension, smoking status, diabetes mellitus, and maximal aortic diameter where appropriate.

## Data Availability

The datasets generated and/or analyzed during the current study are not publicly available because they contain potentially identifiable clinical and biological information and are subject to ethical and privacy restrictions. De-identified data may be available from the corresponding author upon reasonable request, subject to approval by the relevant ethics committee, applicable institutional policies, and data-sharing requirements.
